# Patterns of Recurrence After Postoperative Stereotactic Radiotherapy for Brain Metastases

**DOI:** 10.3390/cancers17091557

**Published:** 2025-05-03

**Authors:** Jeroen A. Crouzen, Anna L. Petoukhova, Martijn Hakstege, Elise E. M. W. van Schaik, Rishi D. S. Nandoe Tewarie, Rob J. A. Nabuurs, Maaike J. Vos, Melissa Kerkhof, Thijs van der Vaart, Johan A. F. Koekkoek, Rogier E. Hagenbeek, Fatih M. Yildirim, Lisette M. Wiltink, Noëlle C. M. G. van der Voort van Zyp, Mandy Kiderlen, Marike L. D. Broekman, Mirjam E. Mast, Jaap D. Zindler

**Affiliations:** 1 Department of Radiation Oncology, Haaglanden Medical Center, Lijnbaan 32, 2512 VA The Hague, The Netherlands; j.crouzen@haaglandenmc.nl (J.A.C.); n.van.der.voort.van.zyp@haaglandenmc.nl (N.C.M.G.v.d.V.v.Z.); m.kiderlen@haaglandenmc.nl (M.K.); m.mast@haaglandenmc.nl (M.E.M.); 2 Department of Medical Physics, Haaglanden Medical Center, Lijnbaan 32, 2512 VA The Hague, The Netherlands; a.petoukhova@haaglandenmc.nl; 3 Department of Neurosurgery, Haaglanden Medical Center, Lijnbaan 32, 2512 VA The Hague, The Netherlands; r.nandoe.tewarie@haaglandenmc.nl (R.D.S.N.T.); r.nabuurs@haaglandenmc.nl (R.J.A.N.); m.broekman@haaglandenmc.nl (M.L.D.B.); 4 Department of Neurology, Haaglanden Medical Center, Lijnbaan 32, 2512 VA The Hague, The Netherlands; m.vos@haaglandenmc.nl (M.J.V.); m.kerkhof@haaglandenmc.nl (M.K.); t.van.der.vaart@haaglandenmc.nl (T.v.d.V.); 5 Department of Neurology, Leiden University Medical Center, Albinusdreef 2, 2333 ZA Leiden, The Netherlands; j.a.f.koekkoek@lumc.nl; 6 Department of Radiology, Haaglanden Medical Center, Lijnbaan 32, 2512 VA The Hague, The Netherlands; r.hagenbeek@haaglandenmc.nl (R.E.H.); f.yildirim@haaglandenmc.nl (F.M.Y.); 7 Department of Radiotherapy, Leiden University Medical Center, Albinusdreef 2, 2333 ZA Leiden, The Netherlands; l.m.wiltink@lumc.nl; 8 Department of Radiotherapy, The Netherlands Cancer Institute, Plesmanlaan 121, 1066 CX Amsterdam, The Netherlands; 9 Department of Neurosurgery, Leiden University Medical Center, Albinusdreef 2, 2333 ZA Leiden, The Netherlands; 10 Department of Cell and Chemical Biology, Leiden University Medical Center, Albinusdreef 2, 2333 ZA Leiden, The Netherlands; 11 Department of Radiation Oncology, HollandPTC, Huismansingel 4, 2629 JH Delft, The Netherlands

**Keywords:** brain metastases, radiosurgery, meningeal carcinomatosis, neurosurgery

## Abstract

Background/Objectives: Neurosurgical resection is the standard treatment for large brain metastases (BMs). Postoperative stereotactic radiotherapy (SRT) is used to reduce local recurrence (LR) but does not always prevent leptomeningeal disease (LMD). This study aims to analyze patterns of tumor recurrence and to identify opportunities for the further improvement of treatment efficacy. Methods: We included 147 patients who underwent resection and SRT for BMs. The distance between the resection cavity target volume and the new tumor growth was calculated. Cox regression analyses were used to assess associations of LMD with various patient characteristics. Results: Median survival after postoperative SRT was 14 months (IQR 6–30) with a 3-year actuarial survival rate of 21%. LR occurred in 20/147 patients (14%). After total resection, LR occurred in 21% of patients after 3 years of follow-up compared to 36% after subtotal resection. Marginal LR occurred in 5/147 patients (3%). LMD was found in 21/147 patients (14%; 3-year actuarial rate, 26%), and it was found more commonly in patients with resected cerebellar metastases (23%; 3-year actuarial rate, 46%) compared to those with cerebral metastases (11%; 3-year actuarial rate 17%) (HR 2.54, 95% CI 1.07–6.04, *p* = 0.034). Conclusions: This study examined patterns of recurrence after postoperative radiotherapy and its implications for radiation dose, radiation field size, and treatment sequence. Local control was high after total resection. Radiation field size appeared adequate given the low incidence of marginal recurrences. Patients with cerebellar metastases showed an increased risk of LMD, underscoring the need for preventive measures, particularly preoperative SRT.

## Simple Summary

Postoperative stereotactic radiotherapy for brain metastases is used to prevent local recurrence and leptomeningeal disease. The aim of this retrospective study was to analyze patterns of tumor recurrence after this treatment. In a population of 147 patients with brain metastases, treated with postoperative stereotactic radiotherapy, we found that high local control rates (79% after 3 years) were achieved after total resection. A higher treatment dose may improve local control rates further, especially after subtotal resection (64% after 3 years). Radiation field size appeared sufficient due to the low levels of local tumor recurrence (3%) in the margins of the radiation field. Leptomeningeal disease most commonly occurred after the treatment of cerebellar metastases. Novel treatment modalities such as preoperative stereotactic radiotherapy may reduce the likelihood of leptomeningeal disease, especially in high-risk patients.

## 1. Introduction

Neurosurgical resection is the standard treatment for brain metastases (BMs) with a maximum diameter of at least 2.5–3 cm [1,2]. The resection of BMs without radiotherapy is associated with higher rates of local recurrence (LR) compared to resection with adjuvant radiotherapy [3]. Therefore, the resection cavity is usually treated with targeted or stereotactic radiotherapy (SRT).

Despite these treatment options, the LR of BMs occurs in approximately 10–30% of patients 1 year after treatment [4,5,6]. LR manifests as tumor regrowth in resection cavities previously deemed tumor-free or as the growth of subtotally resected lesions. LR is further classified as infield recurrence, which is when a new lesion or volume enlargement appears within the area that received radiation treatment (planning target volume; PTV), or as marginal, i.e., on the edge of the PTV and expanding beyond the PTV. With infield LR, it is implied that the radiation dose and fractionation have been inadequate in preventing tumor growth. In cases of marginal LR, an expanded clinical target volume (CTV)–PTV margin might be necessary to treat tumor cells adjacent to the resection cavity. To improve treatment protocols, it is essential to determine recurrence patterns in patients treated with postoperative SRT.

Leptomeningeal disease (LMD) is a common complication observed in BM patients who were treated with resection followed by SRT [7,8]. LMD is a fatal complication of metastasized cancer, characterized by disease progression from the brain parenchyma into the arachnoid, pia mater, and cerebrospinal fluid (CSF). It is theorized that one of the factors contributing to the risk of LMD after surgery is neurosurgical manipulation, which may cause tumor cell spillage into the CSF space [9,10]. As a result, disseminated tumor cells can no longer be targeted with postoperative SRT.

After LMD diagnosis, survival is generally poor, with a median survival of 3–4 months [11,12,13]. Effective treatment methods for LMD are lacking, although tentative results from novel systemic therapies for breast cancer, non-small-cell lung cancer (NSCLC), and melanoma show some potential [14,15,16,17]. This makes it essential to identify the patients at risk of developing LMD and explore optimal treatment strategies to decrease the incidence of LMD.

The purpose of this study is to analyze the patterns of local tumor recurrence and to identify the risk factors for LMD after postoperative SRT.

## 2. Materials and Methods

### 2.1. Patient Selection

Eligibility criteria for this study included at least one BM treated with linear accelerator (linac)-based SRT after resection between 2010 and 2022 at the Haaglanden Medical Center. Patients with a history of LMD, previous cranial RT, or previous neurosurgery were excluded, as were patients whose radiological follow-up was missing. Data were retrospectively collected from electronic health records. In total, 147 patients with 277 BMs were identified and included. In the 55 patients with multiple metastases, the non-resected metastases and the resection cavity were treated with SRT. In 6/55 patients with >1 BM, only the largest of the non-resected BMs were treated with SRT. In these six patients, the remaining punctiform lesions were monitored during follow-up and could be treated with RT in the case of progression. Systemic treatments, such as chemotherapy, targeted therapy, and immunotherapy, were used after RT in these six patients. In five patients, a resection was performed of two adjacently located metastases rather than one.

### 2.2. Radiological Characterization

All BMs were diagnosed with MRI scans using T1-weighted gadolinium-enhanced images. The CTVs were delineated by an experienced radiation oncologist based on the area of contrast enhancement on pre- and postoperative T1-weighted MRI. The PTV was determined as the CTV with margins of 0, 1, or 2 mm. CTV-PTV margins were 2 mm prior to 2015 and 1 mm from 2017 onward. No CTV-PTV margins were utilized between 2015 and 2016, as well as when the PTV was too close to the brainstem. A quality assurance (QA) framework was utilized for MRI sequences of 1.5 T. Moreover, T1-weighted contrast-enhanced volumetric-interpolated breath-hold examination (VIBE) was used to reduce MRI distortions for the delineation of BMs. Patients were treated with a dose schedule based on the PTV size, extent of resection, and preference of the physician. Most patients were treated with three fractions, but a single-fraction SRT was available when, for example, it was necessary to minimize the delay in initiating the systemic treatment. In rare cases, a schedule of 13 × 3 Gy was given in order to reduce toxicity in critical organs. All patients were irradiated with a dedicated linac-based SRT machine, including a robotic couch: between 2010 and 2016 with a Novalis Classic linac (BrainLAB AG, Feldkirchen, Germany) and from 2016 onward with a Versa HD linac (Elekta, Stockholm, Sweden).

### 2.3. Treatment Outcomes

Following resection and SRT, patients underwent MRI scans (including T1-weighted contrast-enhanced, T2-weighted, and dynamic susceptibility contrast (DSC) perfusion-weighted images) every three months until death or the initiation of best supportive care. Adverse findings on these scans included (radiological signs of) radionecrosis, LR, and regional recurrence. Differentiation between radionecrosis and LR was based on histological confirmation, when available. Alternatively, a multidisciplinary tumor board reviewed all cases, integrating information from the clinical course and radiological features to reach consensus on whether it was radionecrosis or LR. The signs suggestive of radionecrosis were progression after initial tumor shrinkage in the irradiated area, the absence of hyperperfusion, increased gadolinium contrast uptake, increased peripheral edema on T2-weighted images, and central hypo-intensity. Regional recurrence referred to new solid tumors outside of the resection cavity area, including dural (non-leptomeningeal) metastases.

LR was categorized as infield or marginal. Infield LR was defined as a new lesion or volume enlargement within PTV boundaries. Marginal LR was defined as volume expansion beyond the PTV boundaries. For each marginal recurrence, it was determined how much the CTV-PTV margin should have been expanded to include the areas where tumor growth was found.

LMD was confirmed through CSF cytology and/or cerebral/spinal MRI showing new, abnormal leptomeningeal enhancement around the brain, spinal cord, or cauda equina visible on T1 gadolinium-enhanced images. The signs suggestive of LMD from CSF analysis included malignant cells, along with an elevated white blood cell count, increased protein levels, and low glucose levels. LMD was seen as probable in cases without positive CSF cytology but with typical radiological and clinical signs, in accordance with Le Rhun et al. [18]. Patients without these signs were not scored as positive for LMD in these analyses.

### 2.4. Statistical Analyses

The overall survival was calculated from the first day of SRT until the date of death, or the date the patient was last known to be alive, and was estimated using the Kaplan–Meier method. The time to LR and LMD was calculated from the first date of SRT until the date of LR/LMD diagnosis or until the last moment of follow-up or death. Kaplan–Meier curves were used to analyze the risk of LR and LMD. A log-rank test was used to assess statistically significant differences in LR rates between patients with totally and with subtotally resected tumors on postoperative MRI. A *p* value of ≤0.05 (two-sided) was considered significant.

Independent variables were investigated for their association with LMD using Cox regression analyses. The characteristics included in these analyses were the following: sex, age at BM diagnosis, Karnofsky Performance Status at BM diagnosis, primary tumor pathology, the number of lesions, the maximum preoperative diameter of resected BMs, the presence of extracranial metastases, the location of resected BMs (supra- or infratentorial), the proximity of resected BMs to CSF space, resection method, the extent of resection on postoperative MRI (total or subtotal), systemic therapy within 2 months of SRT, and radiation fractionation. Proximity between the resected BMs to CSF space was categorized as follows: separated if the tumor was entirely surrounded by brain parenchyma, or contact/involved if the tumor was in direct contact with the pia mater or ventricle wall without any intervening brain tissue [19].

## 3. Results

**Figure A1:**
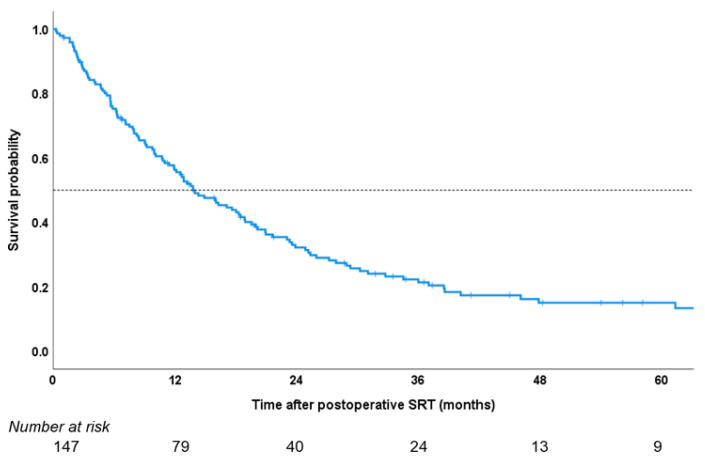
Survival probability after postoperative SRT.

The median age at BM diagnosis was 62 years (range 28–83). The median time between surgery and SRT was 39 days (IQR 31–53). At the time of BM diagnosis, 67 patients (46%) had extracranial metastases. Most patients (63%) had one (resected) tumor at the time of SRT. The most common primary tumor was NSCLC adenocarcinoma (40%). The majority of patients (84%) were treated with postoperative SRT in three fractions. Most patients (124; 84%) had one or two BMs at the time of treatment, while two (1%) had more than ten. In these cases, not all punctiform lesions were treated with SRT. Patient characteristics are presented in Table 1. The median survival after SRT was 14 months (IQR 6–30) (Appendix A, Figure A1). The actuarial survival rates were 56% at 1 year, 32% at 2 years, and 21% at 3 years.

**Figure 1:**
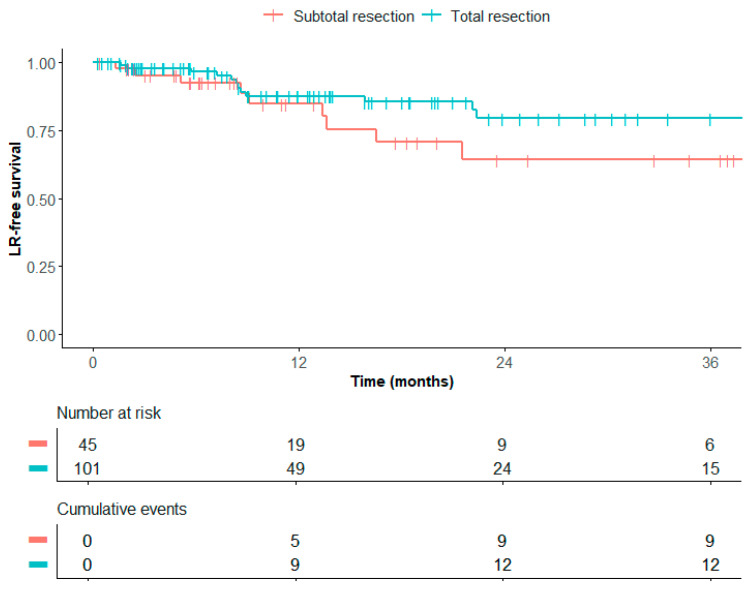
Local recurrence-free survival after subtotal and total resection.

LR occurred in 20/147 patients (14%) of the entire cohort. The median time between LR and LMD was 4 months (range 0.5–13 months). In patients with no trace of residual disease on postoperative MRI, LR rates were 12% after 1 year and 21% after 3 years. In patients with subtotally resected BMs, LR rates were 15% after 1 year and 36% after 3 years (Figure 1). A log-rank test showed no significant difference between these groups (*p* = 0.13). LR was present in 4/21 patients (19%) who later developed LMD.

Of the local recurrences, 15/20 were located infield, and 5/20 were marginal. In the latter five patients, the median distance between the original PTV around the resection cavity and the outer edge of the new marginal tumor growth was 3.1 cm (range 2.3–4.1). The treatment for LR consisted of re-SRT (11/20; 55%), re-resection (6/20; 30%), or whole-brain radiotherapy (3/20; 15%) (Table 2).

The crude incidence of LMD was 21/147 patients (14%), with actuarial rates of 9% at 6 months, 14% at 1 year, 18% at 2 years, and 26% at 3 years. All patients with LMD underwent cerebral/spinal MRI for diagnosis. CSF cytology confirmed the (radiological) diagnosis in 7/21 patients (33%). In one patient, CSF cytology was negative, but the chemical analysis of CSF was indicative of LMD. MRI later confirmed the diagnosis. In the six patients with multiple BMs, where not every BM was treated with SRT, one (16%) developed LMD. After LMD diagnosis, the median survival time was 4 months (IQR 1–6). The treatment for LMD included best supportive care (29%), whole-brain radiotherapy (43%), and systemic treatment (38%) (Table 2). Those who only received best supportive care had a median survival time of 11 days (IQR 7–22) after LMD diagnosis, while those who underwent any therapy for LMD had a median survival time of 5 months (IQR 3–8).

**Figure 2:**
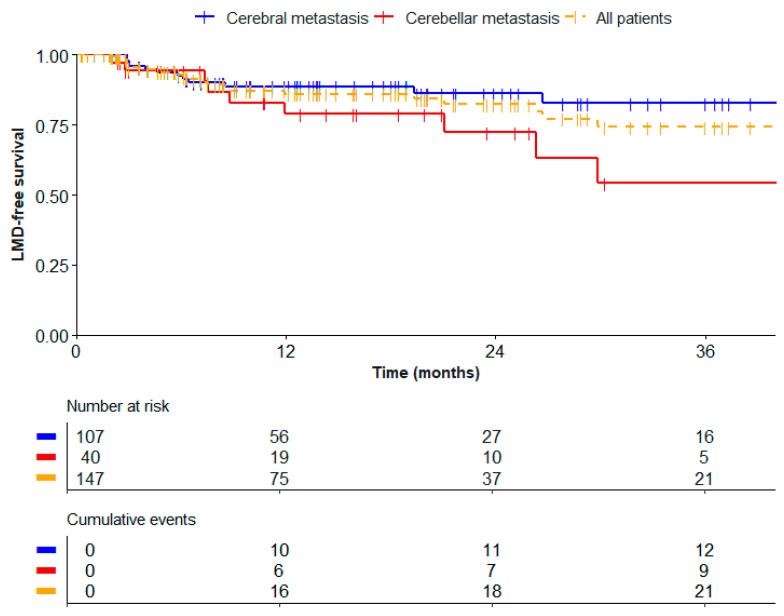
LMD-free survival after postoperative SRT.

In the univariate Cox regression analyses, the cerebellar location of a BM was significantly associated with the development of LMD (HR 2.54, 95% CI 1.07–6.04, *p* 0.034). LMD occurred in 9/40 patients with cerebellar metastasis (23%). The actuarial incidence was 6% at 6 months, 11% at 1 year, 18% at 2 years, and 46% at 3 years. LMD occurred in 12/107 patients with cerebral metastasis (11%). The actuarial incidence was 8% at 6 months, 11% at 1 year, 14% at 2 years, and 17% at 3 years (Figure 2). No other characteristics, including the number of lesions and extent of resection, were significantly associated with the development of LMD (Table 3), so no multivariable Cox regression analysis was performed.

Regional tumor recurrence occurred in 80/147 patients (54%) of the entire cohort and in 16/21 patients (76%) who later developed LMD. The treatment for regional recurrence consisted of SRT (43/80; 54%), resection (6/80; 8%), or whole-brain radiotherapy (20/80; 25%) (Table 2). Symptomatic radionecrosis occurred in 16/147 patients (11%) with a 1-year actuarial incidence of 16%. All patients with symptomatic RN were treated with corticosteroids, three of whom (19%) received further treatment with bevacizumab and one of whom (6%) underwent a resection for radionecrosis.

## 4. Discussion

In this study, we show high local control rates in BMs treated with postoperative SRT. Infield LR (11%) was more commonly seen compared to marginal LR (3%). This pattern of recurrence suggests that ensuring optimal dose delivery to the PTV is more relevant than expanding CTV-PTV margins to prevent LR. We found that patients who had undergone a subtotal resection were at an increased risk of LR (21%, 3 years after total resection versus 36%, 3 years after subtotal resection), although this effect was not statistically significant. To reduce LR rates, strategies such as an increased radiation dose could be considered [20]. These strategies should especially be employed in patients with larger (preoperative) tumor volume or after subtotal resection. However, the potential for lower LR rates should be balanced against the potentially higher risk of toxicities, such as radionecrosis. Further prospective studies are required to validate these strategies. Currently, a randomized trial is investigating the impact of hypofractionation on LR and radionecrosis (ClinicalTrials.gov identifiers: NCT05346367) [21].

The low rate of marginal tumor growth suggests that current radiation field sizes with CTV-PTV margins of 0–2 mm are generally adequate. These margins are the most often reported in the literature [22]. When marginal tumor growth did occur, it extended well beyond the original PTV. The median distance from PTV to its furthest continuous extent was 3.1 cm. An expansion of the CTV-PTV margin between 1 and 5 mm would have been insufficient to encompass the area where marginal tumor growth occurred. The potential advantage of reduced marginal LR rates through wider margins would therefore likely be limited and would not outweigh the potential side effects from such an extensive expansion.

Furthermore, the tumor location was found to be the only variable significantly associated with the development of LMD after postoperative SRT. The crude incidence of 14% with an actuarial incidence of 18% after two years is in line with the incidence between 10 and 20% described in the literature [8,23,24,25,26,27]. Our finding that LMD is more common after the treatment of cerebellar metastases compared to cerebral metastases is also consistent with other studies [28,29,30,31,32]. A number of studies suggest that the close proximity of cerebellar BMs to the large CSF spaces in the posterior fossa, like the cisterna magna, might be a reason for the increased risk of LMD in these patients [31,32]. These CSF spaces could act as reservoirs for tumor cell spillage during surgery, which may increase the risk of LMD. The comparatively small subarachnoid space, which contains CSF, surrounds superficial cerebral metastases, so intraoperative tumor spill may be less likely to occur here. The close proximity of BMs to any CSF space could be theorized to lead to an increased risk of LMD [33]. In our study, patients with BMs close to CSF spaces were found to be at an increased risk of LMD, although this effect was not statistically significant (HR 2.16, 95%CI 0.72–6.46, *p* 0.17).

Neurosurgery with postoperative SRT has been associated with an increased risk of the development of LMD compared to SRT only [7,8]. This may be due to selection bias, where larger and more aggressive tumors are treated in healthier patients with a longer life expectancy and thus have more time to develop LMD. Limiting intraoperative tumor dissemination remains challenging due to a limited number of known surgery-specific risk factors. One previously reported risk factor is tumor spillage after a piecemeal resection method rather than en bloc [19,34]. Likewise, intraoperative ventricle violation has been associated with an increased risk of LMD [35]. Previous studies have reported several other risk factors for developing LMD, including multiple BMs at baseline, younger age, large preoperative tumor size, the presence of extracranial metastases, and breast cancer as the primary tumor location [7,8,23,28,29,32,33,34,36,37,38,39,40]. Our study found an association between some of these factors and an increased risk of LMD, but only the tumor location was statistically significant. This may be due to a smaller study cohort with fewer total events compared to other studies or due to the limited number of patients from specific subgroups, such as breast cancer patients. A larger cohort could potentially have shown more factors significantly associated with LMD.

Since patients with cerebellar metastases are at an increased risk of developing LMD, this group is likely to benefit most from alternative treatment strategies aimed at mitigating this risk, such as preoperative SRT. Nevertheless, patients with cerebral metastases were three times more prevalent than patients with cerebellar metastases in this study, accounting for over half of the LMD cases. Therefore, studies on treatments like preoperative SRT ought to include patients with cerebral metastases as well.

The relatively high prevalence of LMD highlights the importance of finding alternative treatment strategies to prevent the detrimental effects of LMD. It is hypothesized that preoperative SRT enables the sterilization of tumor cells before the intraoperative spillage of malignant cells into the CSF can occur [41,42,43]. Retrospective studies suggest that preoperative SRT is associated with a lower risk of LMD compared to postoperative SRT, while there is no difference compared to WBRT [10,44,45]. The largest comparative studies were performed by Patel et al. A retrospective study from this group (n = 102) showed no difference in overall survival, local/distant recurrence, and LMD rates between preoperative SRT and postoperative WBRT [44]. In another study (n = 180), the same group found higher rates of LMD (17% versus 3%, *p* = 0.01) two years after postoperative SRT compared to preoperative SRT [46].

Another benefit of preoperative SRT is the decreased dose exposure in healthy brain tissue due to a more clearly defined PTV compared to the postoperative situation [45]. This might also reduce LR due to the fact that it becomes less difficult to determine where there is tumor activity and where the radiation dose should be delivered. Likely due to the smaller PTV, the incidence of radionecrosis is lower after preoperative SRT [43,44,47]. Additionally, there are no increased risks of complications, such as delayed wound healing, following preoperative SRT and surgery [9,10,48]. Currently, several prospective studies comparing pre- and postoperative SRT are ongoing in Europe and North America, including four randomized trials (ClinicalTrials.gov identifiers: NCT03741673, NCT05124236, NCT04474925, NCT03750227).

This study has several limitations that should be considered when interpreting the results. The results of this retrospective study need to be confirmed by prospective studies to reduce the effect of selection bias. While the cohort size was generally sufficient, a larger cohort would have increased the study’s power to detect statistically significant differences, such as the impact of the extent of resection on LR. A larger cohort size would have allowed for further subgroup analyses as well. Additionally, the diagnosis of LMD was based on radiological findings in the majority of cases rather than confirmation from CSF cytology. Patients with suspected LMD could be at the point of their disease where relatively invasive procedures such as lumbar puncture are deemed unwanted to not further inconvenience the patient, which explains why CSF cytology was not always available. The radiological diagnosis of LMD can be challenging, leading to interobserver variability in interpretation between radiologists and potentially inconsistent findings. When the diagnosis was uncertain, an experienced neuroradiologist was consulted to reassess the imaging. CSF cytology can likewise produce false-negative results, which may lead to the underestimation of the true incidence of LMD. The EANO-ESMO LMD guideline established standardized diagnostic criteria and a level of evidence for LMD [18]. The diagnostic criteria from this guideline were used in our study to distinguish between cases where LMD was confirmed/probable and cases where LMD was only “possible”. Despite these limitations in diagnostic methods, our observed incidence rates are comparable to those reported in the other literature [8,23,24,25,26,27]. Furthermore, the groups were unevenly distributed in terms of BM location, with only 27% of BMs located in the cerebellum. This explains the relatively wide range of the 95% confidence interval in this group (Table 3). Lastly, LMD may have been missed in patients who died outside of a hospital setting. Despite the aforementioned limitations, this study has several strengths, including a relatively homogenous patient population, a relatively long follow-up interval, consistent treatment approaches, and regular MRI follow-up. The findings of this study are consistent with those of previous research [8,23,24,25,26,27].

## 5. Conclusions

This study describes patterns of recurrence after postoperative radiotherapy. Local control was high after resection but may have improved with an increased radiation dose. Radiation field size appeared adequate given the relatively low incidence of marginal recurrences. Cerebellar metastases are at an increased risk of LMD compared to cerebral metastases, underscoring the importance of exploring preventive measures, particularly preoperative SRT, to mitigate the risk of LMD in these patients.

## Figures and Tables

**Table 1:** Patient characteristics.

Characteristic	Overall (n = 147)
Sex	
Male	55 (37%)
Female	92 (63%)
Age at BM diagnosis	
Median (range)	62 years (28–83)
Karnofsky Performance Status	
100	11 (8%)
90	59 (40%)
80	35 (24%)
70	33 (22%)
60	5 (3%)
Unknown	4 (3%)
Primary tumor histology	
Breast cancer	24 (16%)
NSCLC (adenocarcinoma)	59 (40%)
NSCLC (non-adenocarcinoma)	16 (11%)
Colorectal cancer	11 (8%)
Other	37 (25%)
Number of lesions	
1	92 (63%)
2	32 (22%)
3 or more	23 (15%)
Max preoperative diameter of resected BM	
Median (range), all metastases	38 mm (15–75)
Median (range), supratentorial metastases	38 mm (15–75)
Median (range), infratentorial metastases	36 mm (17–56)
Presence of extracranial metastases	
Yes	67 (46%)
No	80 (54%)
Location of resected BM	
Cerebral (supratentorial)	107 (73%)
Cerebellar (infratentorial)	40 (27%)
Proximity to CSF space	
Contact or involvement	99 (67%)
Separated	43 (29%)
Unknown	5 (3%)
Resection method	
Piecemeal	62 (42%)
En bloc	63 (43%)
Unknown	22 (15%)
Extent of resection	
Total	101 (69%)
Subtotal	45 (31%)
Unknown	1 (1%)
Systemic therapy within 2 months of SRT	
Chemotherapy	37 (25%)
Hormonal therapy	7 (5%)
Targeted therapy	18 (12%)
Immunotherapy	15 (10%)
None	79 (54%)
Unknown	7 (5%)
Radiation fractionation and dose	
1 × 15 Gy	1 (1%)
1 × 18 Gy	9 (6%)
1 × 21 Gy	10 (7%)
3 × 8 Gy	59 (40%)
3 × 8.5 Gy	65 (44%)
7 × 5 Gy	1 (1%)
13 × 3 Gy	2 (1%)

**Table 2:** Salvage treatment options after tumor recurrence.

Treatment Type	Number of Patients
Local recurrence (n = 20)	
Re-SRT	11 (55%)
Re-resection	6 (30%)
Whole-brain radiotherapy	3 (15%)
Regional recurrence (n = 80)	
SRT	43 (54%)
Resection	6 (8%)
Whole-brain radiotherapy	20 (25%)
Leptomeningeal disease (n = 21)	
Whole-brain radiotherapy	9 (43%)
Systemic treatment	8 (38%)

**Table 3:** Univariate Cox regression analyses of risk factors for LMD after postoperative SRT.

Characteristic	HR (95% CI, *p*)
Sex	
Male	1.0 (reference)
Female	0.66 (0.28–1.56, 0.34)
Age (years)	1.00 (0.96–1.05, 0.87)
Primary tumor histology	
Breast cancer ^1^	1.0 (reference)
Lung cancer (adenocarcinoma)	2.14 (0.47–9.77, 0.33)
Other	2.25 (0.48–10.5, 0.30)
Number of lesions	
1	1.0 (reference)
2	1.30 (0.50–3.40, 0.59)
3 or more	0.27 (0.04–2.45, 0.27)
Max preoperative diameter of resected BM (mm)	1.02 (0.98–1.06, 0.31)
Presence of extracranial metastases	
Yes	1.0 (reference)
No	0.81 (0.34–1.92, 0.63)
Location of resected BM	
Cerebral (supratentorial)	1.0 (reference)
Cerebellar (infratentorial)	2.54 (1.07–6.04, 0.034)
Tumor proximity to CSF space	
Separated	1.0 (reference)
Contact or involvement	2.16 (0.72–6.46, 0.17)
Resection method	
Piecemeal	1.0 (reference)
En bloc	0.74 (0.30–1.84, 0.51)
Extent of resection	
Total	1.0 (reference)
Subtotal	1.15 (0.46–2.81, 0.76)
Systemic therapy within 2 months of SRT	
Yes	1.0 (reference)
No	0.48 (0.20–1.16, 0.10)
Radiation fractionation	
3×	1.0 (reference)
1×	0.23 (0.03–1.72, 0.15)

^1^ breast cancer was used as a reference due to higher LMD rates in the literature (see Section 4).

## Data Availability

The original contributions presented in this study are included in the article. Further inquiries can be directed to the corresponding author.

## Abbreviations

The following abbreviations are used in this manuscript:

BM(s)	Brain metastasis/metastases
CSF	Cerebrospinal fluid
CTV	Clinical target volume
DSC	Dynamic susceptibility contrast
IQR	Interquartile range
LMD	Leptomeningeal disease
LR	Local recurrence
MRI	Magnetic resonance imaging
NSCLC	Non-small-cell lung cancer
PTV	Planning target volume
QA	Quality assurance
SRT	Stereotactic radiotherapy
VIBE	Volumetric-interpolated breath-hold examination
